# Validation of the english version of the Multidimensional Mentalizing Questionnaire (MMQ)

**DOI:** 10.1186/s40359-024-01837-z

**Published:** 2024-06-12

**Authors:** Germano Vera Cruz, Lucien Rochat, Magdalena Liberacka-Dwojak, Monika Wiłkość-Dębczyńska, Riaz Khan, Yasser Khazaal

**Affiliations:** 1 Department of Psychology, CRP-CPO, University of Picardie Jules Verne, Amiens, UR 7273 France; 2 Department of Mental Health and Psychiatry, Specialized Facility in Behavioral Addiction ReConnecte, University Hospitals of Geneva, Geneva, Switzerland; 3 Department of Psychology, Kazimierz Wielki University, Bydgoszcz, Poland; 4 Frontier Medical College, Bahria University Islamabad, Islamabad, Pakistan; 5 Addiction Medicine, Department of Psychiatry, Lausanne University Hospital and Lausanne University, Lausanne, Switzerland; 6 Research Centre, University Institute of Mental Health at Montreal and Department of Psychiatry and Addiction Montreal University, Montreal, Canada

**Keywords:** Mentalizing, Cognition, Loneliness, Mood, Social functioning

## Abstract

**Background:**

Mentalizing refers to the ability to understand one’s own and others’ mental states. Mentalizing is considered a key component of social cognition and healthy personality development. A multinational assessment tools able to appraise the multidimensional and multifaceted aspects of this complex construct are needed.

**Objective:**

The present study had two aims: (a) validate an English version of the Multidimensional Mentalizing Questionnaire (MMQ, 33 items) which was designed to assess mentalizing based on an integrated and multilevel model of mentalizing; (b) explore the correlational relationships between the six dimensions of the MMQ and a set of sociodemographic, psycho-cognitive, mental health, and socio-functional variables.

**Methods:**

Overall, 1823 individuals (age: 19–76 years old [M = 45; SD = 16]; sex: male = 48.51%, female = 50.57%, non-binary = 0.9%) participated in an online survey. While the participants came from 77 different countries, most of them were residents in UK and USA (95%). Data analytics include confirmatory factorial analysis and Pearson correlations.

**Results:**

The CFA results validated the factorial structure of a 28-items MMQ-English version, with acceptable goodness of fit indices. Regarding the psychometric properties, the MMQ-English version showed good internal reliability and significant positive correlation with another scale designed to assess an analogue construct showing a fair convergent validity. The findings indicated that males, individuals with lower levels of education, lower socio-economic status, depressed, and with a higher score of loneliness are significantly more likely to report poor mentalizing compared with females, individuals with higher education level, greater SES, happier, and with lower scores of loneliness.

**Conclusion:**

The present study validated the English version of the MMQ.

**Supplementary Information:**

The online version contains supplementary material available at 10.1186/s40359-024-01837-z.

## Introduction

Mentalizing (also known as mentalization) is “the ability to understand one’s own and others’ mental states, thereby comprehending one’s own and others’ intentions and affects” [1, p.1]. In other words, this ability or mental activity allows a person to interpret the behaviors of others or their own by referring to the mental states (beliefs, feelings, wishes, thoughts, etc.) which could be at the origin of these behaviors [2]. Mentalizing is related to, but distinct from, theory of mind. In fact, both concepts describe metacognitive processes [3]. However, mentalizing mainly concerns the reflection of cognitive and affective mental states in the context of emotional arousal [3, 4]. In contrast, “theory of mind focuses more specifically on epistemic states such as beliefs, intentions, and persuasions.” [3, p.730].

### Mentalizing in human cognition, behavior, and health

Mentalizing is considered a key component of social cognition and healthy personality development, playing a significant role in people’s ability to communicate, interpersonal functioning and entertaining relationships, as well as for feeling and expressing empathy, regulating their emotions, impulse control, reflective functioning, and experiencing well-being [2, 5, 6].

Particularly, mentalizing deficiency has been found to be associated with poor mental health [5] and psychopathologies states such as borderline personality disorder (BPD [7, 8]. These findings have led to the development of mentalizing-based therapies (MBT [9, 10]) and mentalizing-based educational interventions (MBEI [11, 12]).

### Development of mentalizing measurement instruments

Following the theorization of reflective functioning in the concept of mentalizing in the 1990s, researchers have begun to develop measurement tools [13]. Task-based (e.g., the Affect Task [AT] [13]) and narrative-based (e.g., the Mentalizing Stories for Adolescents [MSA] [14]) mentalizing assessment instruments were specifically developed to be used among children and young adolescents. Interview-based and clinical-report mentalizing screening tools (e.g., Reflective Functioning Scale [RFS] [15] and the Metacognition Assessment Scale [MAS] [16]) were designed to be used among older adolescents (16 + years-old) and adults.

The RFS is a unidimensional screening tool. It assesses individual differences in the ability to mentalize attachment relationships using transcripts of interviews (Adult Attachment Interview). A mentalizing global score is attributed by trained interview codifiers and interpreters on an 11-point scale ranging from anti-reflective to exceptionally reflective [15]. Also based on clinical interviews (Metacognitive Assessment Interview), the MAS overcomes the one-dimensionality problems of the RFS by differentiating metacognitive processes into three dimensions: understanding one’s own mind, understanding others’ minds, and mastery [16]. The first two factors are related to the capacity to reflect upon the self and others’ mental states, while the third factor represents the ability to regulate and control mental states [16]. Albeit highly reliable, the RFS and MAS require time-consuming session transcripts or interviews for assessment and lengthy training to use them correctly [13]. These disadvantages restrict their applicability in large-scale studies involving a relatively large sample of patients, thus limiting their use to highly specialized research and clinical contexts [13].

Mentalizing screening instruments based on self-report measures have been developed to overcome the limitations of tools based on interviews. That is the case of the Reflective Functioning Questionnaire (RFQ [17]) and the Mentalization Questionnaire (MZQ [18]). The RFQ is unidimensional and brief screening measure of reflective functioning, specifically designed to assess severe impairments/imbalances in mentalizing as typically observed in patients presenting features of borderline personality disorder [17]. That is, the RFQ is not suitable to be used among general population, since was not designed to capture the different dimensions of mentalizing, nor to assess the process of mentalizing as it unfolds in social interactions [17].

The MZQ [18] is a multidimensional 15-item self-report measure assessing four dimensions: (a) refusing self-reflection (avoidance of thinking about inner states or a systematic rejection of one’s own feelings combined with the fear of being overwhelmed by them); (b) emotional awareness (lack of perceiving and differentiating one’s own inner states); (c) the psychic equivalence mode (the pre-mentalizing modality of thought in which inner mental states and outer reality are equated and everything appears real, related to an unstable inner representation of relations and a lack of flexibility); and (d) the regulation of affect (the inability to modulate affect, that can produce feelings of helplessness and make people feel threatened by their own feelings). Finally, the Mentalization Imbalances Scale (MIS [19]) is a much more recent clinical-oriented, and multidimensional assessment measure of mentalizing. It has six factors representing different imbalances of mentalizing: cognitive, affective, automatic, external, imbalance toward others, and imbalance toward self.

The five (RFS, MAS, RFQ, MZQ, and MIS [15–19]) mentalizing assessment tools presented above were designed mainly as clinical screening tools to be used among patients with psychopathological states. In the last years, attempts to develop instruments designed to assess the transdiagnostic and continuum components of mentalizing both among a general and clinical population were made. The Mentalization Scale (MentS [20]) assesses mentalizing capacity in three dimensions: self-related mentalization (MentS-S), other-related mentalization (MentS-O), and motivation to mentalize (MentS-M). The Interactive Mentalizing Questionnaire (IMQ [21]) is a 20-item, self-report instrument developed to measure “second order mentalization”, associated with people’s beliefs about how transparent their thoughts are to others, or whether this capacity plays a significant role in social interaction/functioning.

The review above shows that, currently, there is several self-reported instruments designed to assess multiple dimensions of mentalizing. Still, many researchers and clinical practitioners [22–25] continue to express dissatisfaction with the existing self-report instruments, wondering: (a) What do day exactly assess? Do they measure mentalizing ability or a belief about one’s mentalizing abilities? (b) Do these instruments are able to capture the multifaceted nature of the mentalizing, that is, beyond the multidimensional aspect, its multilevel aspect?

A satisfactory answer to the first set of questions would require the development of a “gold standard” task-based mentalizing assessment tool that could serve as a benchmark measure for a given self-report scale validity. To our knowledge, a consensual “golden standard” task-based assessment tool has not been developed yet. In contrast, Gori et al. [22, 23] developed and validated the Multidimensional Mentalizing Questionnaire (MMQ) among a sample of Italian speaking individuals, in an attempt to respond to this need of an enhanced and versatile framework to assess mentalizing in its multifaceted nature. This instrument has 33 items grouped into six dimensions: reflexivity; ego-strength; relational attunement; relational discomfort; distrust; and emotional dyscontrol. These dimensions can be integrated into a multilevel model of mentalizing (see Fig. 1 in Gori et al. [22, 23]), which comprise two dynamics: (a) in one hand, *Good mentalizing* (cognitive-affective integration [first level]; internal-external openness [second level]; associated to the flexibility, ego-strength, and relational attunement dimensions [third level]); (b) *Bad* (poor) *mentalizing* (cognitive-affective split [first level]; internal-external closure [second level]; associated to relational discomfort, distrust, emotional dyscontrol [third level]).

**Fig. 1:**
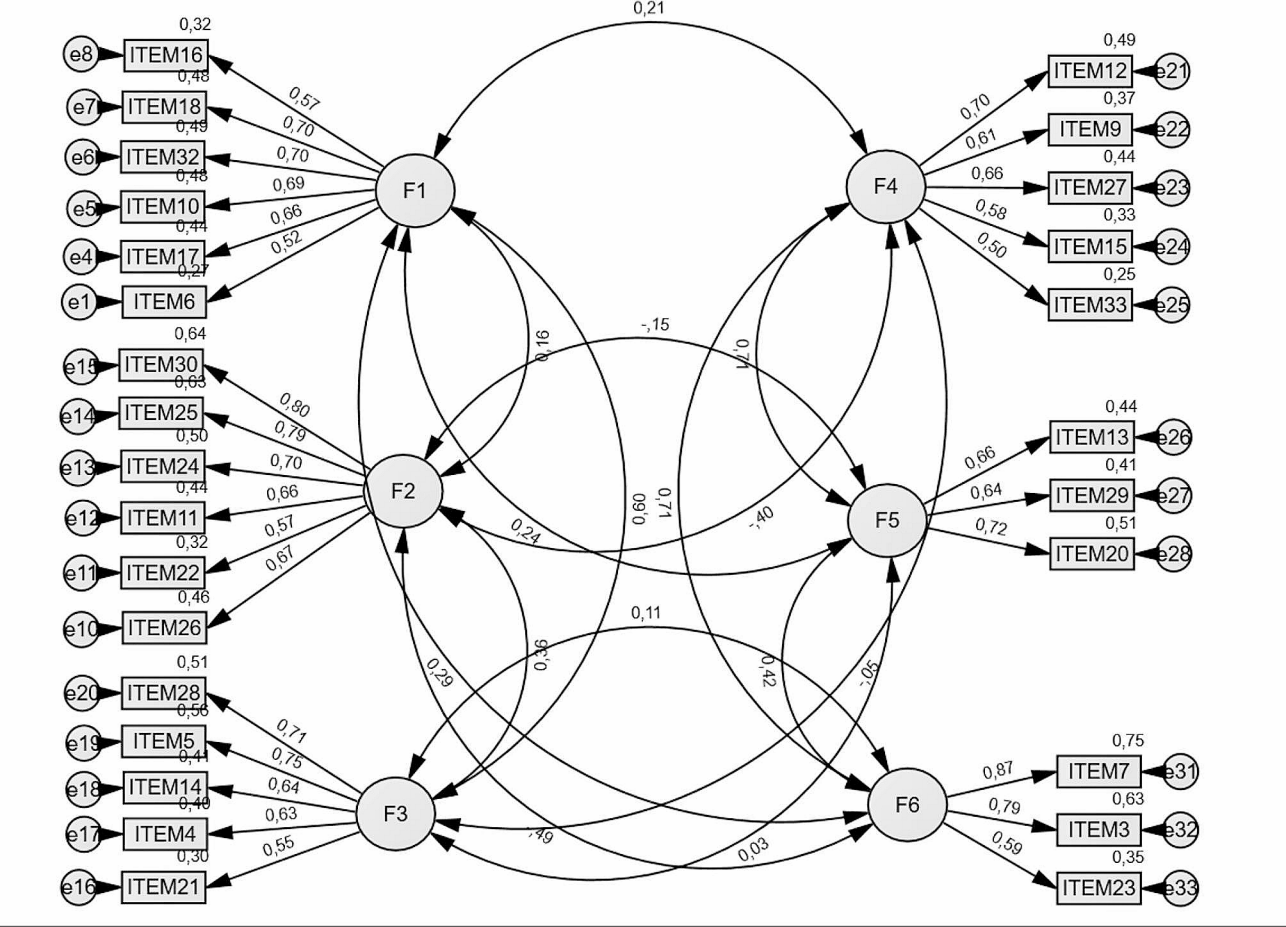
The CFA path diagram of the first improved model (model-2, 28 items) with the standardized estimates Confirmatory factor analysis of the questionnaire. The rectangles represent the different items, and the ellipses represent the factors (F1 = reflexivity; F2 = ego-strength; F3 = relational attunement; F4 = relational discomfort; F5 = distrust; F6 = emotional dyscontrol). The values on the arrow linking the six factors between them are the correlations. The values on the arrow linking each of the six factors to the corresponding items are the factors loadings (standardized estimates). The factor loadings indicate how well each item is representative of its unobservable construct (factor). Its values go from 0 to 1. The values on top of each rectangle are the square of the standardized factor loadings; they give the proportion of the explained variance (R^2^) in each item, which indicates how much of the variance in the item is explained by the unobserved construct. If a standardized factor loading value is greater than 0.70 or explains at least half (0.50 = 50%) of the variance in the item, then the corresponding item is important in explaining the unobserved construct it belongs to. The small rounds are the error terms (measurement errors for each item), and the values on the arrow linking the error terms are the covariances established to improve the model fitting metrics

According to Gori et al. [22, 23], the MMQ showed good psychometric properties such as ability to discriminate between general populations and clinical populations and supports the association between poor mentalizing and psychopathology, underlying the centrality of the mentalizing construct in different forms of psychopathology. Moreover, the six dimensions of the MMQ were significantly correlated with a set of neuro-psychological and socio-functional constructs such as *alexithymia* (difficulty identifying feelings and distinguishing between feelings and bodily sensations in emotional activation, difficulty in the verbal expression of emotions, externally oriented thinking), *impulsiveness* (attentional impulsiveness, motor impulsiveness, non-planning impulsiveness), *attachment styles* (secure attachment, preoccupied attachment, avoidant attachment, unresolved attachment), *personality traits* (extroversion and agreeableness), and *self-esteem*. However, this original version of the MMQ [22, 23] was validated in Italian and among a relatively homogenous sample in terms of sex and origin.

### The present study purpose

Given the psychometric qualities and the potential utility of the original version of the MMQ [22, 23], the present study had two aims: (a) assess the factor structure and the psychometrics properties of the MMQ-English version among a relatively large sample of multinational participants; (b) explore the correlations between the six dimensions of MMQ and a set of sociodemographic, psycho-cognitive, mental health, and socio-functional variables, namely: (i) the participants’ sociodemographic characteristics, (ii) the participants’ overall score on a theory of mind scale, (iii) the participants score on a happiness-depression scale, and (iiii) the participants score on a loneliness scale.

## Methods

### Participants

Overall, 1823 individuals participated in the survey, answering an online questionnaire. The participants were resident in United Kingdom = 1482(77.2%), United States = 342(17.8%), Ireland = 31(1.6%), Australia = 29(1.5%), Sweden = 24(1.3%), New Zealand = 12(0.6%). They had the different nationalities across 27 different European countries = 1466(76.1%), 2 Nord-American countries = 307(16%), 14 Asian countries = 51(2.7%), 8 African countries = 31(2%); 2 Ocean countries = 34(1.8%); 8 Latino-America = 13(0.8%), and 5 Middle east countries = 11(0.8%).

### Recruitment and sampling

The recruitment of study participants was conducted anonymously, using the Prolific services [26]. Prolific is private company specialized on research data collection and offering a service that includes a guarantee regarding the quality of the data. They have a “reservoir” of individuals willing to participate on scientific online research surveys. Prolific has been described as possessing some advantages over other similar platforms, including that it is exclusively dedicated to research studies, and its participants are more ethnically and geographically diverse [27]. Following quality checks, we found no missing values and no significant outliers in the data. Thus, there was no need to remove any observation from the datas.

### Data collection material

The data was collected via online questionnaire, a task outsourced to Prolific, as explained above. The questionnaire included:

#### Socio-demographic questions (7 variables)

It included: age, sex (male, female), relationship status (single, in relationship), level of education (measured in terms of years of schooling), and socio-economic status (SES; low, intermediate, high).

#### The Multidimensional Mentalizing Questionnaire (MMQ [22, 23])

It is a 33-item self-report measure covering different core mentalizing constructs on four different axes: (a) cognitive–affective; (b) self–other; (c) outside–inside; and (d) explicit–implicit. The Likert response scale ranges from 1 (Not at all) to 5 (A great deal).

#### The Four-Items Mentalising Index (FIMI [28])

It is a self-report measure to assess an individual’s mentalizing (or Theory of Mind) ability, which in this particular case means the ability to understand and infer the cognitions of others, including their perceptions, intentions, and beliefs. Item sample: “I find it easy to put myself in somebody else’s shoes”. Items 1, 3, and 4 are scored on a scale from 1 (Strongly disagree) to 4 (Strongly agree). Item 2 is reverse-scored. Total scores range between 4 and 16. Before data analysis, we reversed-coded the relevant items so that the greater the overall FIMI score the greater the theory of mind abilities. The scale internal consistency (Cronbach’s alpha coefficient [α]) was 0.77.

#### The Short Depression-Happiness Scale (SDHS [29])

The SDHS includes six items assessing depression (e.g., “I feel dissatisfied with my life”) or happiness (e.g., “I feel happy”). For each item, the response ranges from 1 (Never) to 4 (Often). In the current study, the scoring of the happiness items was reversed to ensure that higher scores indicate depressive mood, and lower scores indicate happiness. The scale’s Cronbach α in the current study was 0.90.

#### The UCLA 3-item loneliness scale [30]

An example item is: “How often do you feel that you lack companionship?”. Participants are required to respond using a 3-point scale: Hardly ever (1); Some of the time (2); Often (3). The scale’s Cronbach α in the current study was 0.85.

### Ethics

The study was carried out in accordance with the Declaration of Helsinki (World Medical Association [WMA] [31]). Participants gave digital informed consent for their survey contribution. That is, for the participants to be allowed to participate in the anonym online survey, they were required to read a written consent form and sign it electronically by clicking a consent button. Participation was voluntary and restricted to those aged ≥ 18 years. All data was anonymously collected. The ethical approval no. KB 390/2022 was obtained from The Bioethics Committee of the Nicolaus Copernicus University functioning at Collegium Medicum in Bydgoszcz, Poland.

### Statistical analyses

First, we conducted a descriptive analysis (range, mean [M], standard deviation [SD], skewness, and kurtosis).

Second, to assess the appropriateness of the data for factorial analyses (assumption of adequacy and sphericity, we applied the Kaiser–Meyer–Olkin test [KMO]) and the Bartlett’s test), respectively.

Third, we conducted confirmatory factor analysis (CFA), applying the maximum likelihood estimation (MLE), to test the dimensionality and construct validity of the MMQ. The MLE has proven to be effective even for categorical variables, such as those obtained from our Likert-type scale data, especially in relatively large samples [32, 33]. To evaluate model fit, we used the indices most recommended by statisticians [34], including the Comparative Fit Index (CFI), the Tucker Lewis fit index (TLI), Standardized Root Mean Square Residual (SRMR), and the Root Mean Square Error of Approximation (RMSEA). According to Goretzko et al. [34] and Hu and Bentler [35], cutoff values for CFI and TLI ≥ 0.95 and ≥ 0.90 indicate respectively good and acceptable fit; a SRMR ≤ 0.08, and a RMSEA ≤ 0.06 indicate good model fit. In addition, we conducted a test of factorial structure invariance between the sample of two countries (UK vs. USA) representing 95% of the total sample.

Fourth, we assessed the internal consistency (reliability) of the factorial structure by computing the Cronbach’s alpha coefficient (α) of each dimension, as well as by calculating the Corrected Item-total Correlation. Cronbach’s alpha and Corrected Item-total Correlation were used as measures of reliability. A α ≥ 0.8 (≥ 0.7) indicating good (acceptable) internal consistency [36, 37]. A good Corrected Item-total Correlation is set at *r* (correlation coefficient) ≥ 0.30 [36, 37].

Fifth, to test the convergent validity, we conducted correlations analysis between the participants total score on MMQ-English version and the participants total score on the FIMI (assessing theory of mind).

Finally, to estimate the relationships between the six dimensions of the MMQ and the sociodemographic, socio-functional, and psycho-cognitive variables, we run correlations (Pearson) analysis. According to Cohen [38], a value of *r* = 0.10, 0.30, and 0.50 indicates a small, medium, large effect, respectively.

The analyses were conducted using IBM SPSS statistics software (version 29) and AMOS statistical software (version 26).

## Results

### Descriptive statistics on the participants sociodemographic variables

The socio-demographic characteristics distribution of the all sample was as follow: Number: 1823; Age: 19–65 years-old (M = 31.66, SD = 6.74); Sex: males = 1155(63.4%), females = 636(34.9%); non-binary = 32(1.8%); Relationship status: single = 900(49.4%), in relation, not married = 567(31.1%), in relation, married = 317(17.4%), divorced = 37(2.0%), widow(er) = 2(0.1%); Socio-economic status: low = 483(26.5%), intermediate = 1265(69.4%), high = 75(4.1%); Ethnicity: White = 79.0%, Asian = 9.4%, Mixed = 4.8%, Other = 2.2%; Educational level (years of schooling): min = 4, max = 27, M = 15.94, SD = 3.08.

### Descriptive statistics of the MMQ 33 items

Table 1 displays the descriptives statistics related to the MMQ 33 items.

**Table 1:** Descriptive statistics of the MMQ (33 initial items grouped into 6 factors) and of the other psychological variables

Factors / Items	Scale/Range	Mean	SD	Skewness	Kurtosis
**Factor 1: Reflexivity**					
1. I often try to explain what is happening to me	1–5	3.15	1.14	-0.31	-0.75
16. I ponder over what happens to me	1–5	3.64	1.06	-0.54	-0.35
18. I often think about why things happen	1–5	3.88	1.00	-0.73	-0.03
32. I’m keen on understanding why certain things happen to me	1–5	3.86	0.93	-0.61	-0.05
10. I’m interested in understanding my mental processes	1–5	3.96	1.02	-0.91	0.31
17. I find beneficial to analyse my behaviour	1–5	3.65	1.04	-0.53	-0.31
31. I am a thoughtful person	1–5	4.12	0.89	-1.00	0.91
8. I am able to reflect on my behaviours	1–5	3.88	0.94	-0.72	0.17
6. Understanding what others feel is crucial in understanding their actions	1–5	3.94	0.92	-0.81	0.37
**Factor 2: Ego-strength**					
30. I am able to cope with difficult situations	1–5	3.51	0.98	-0.40	-0.35
25. I am able to bear the emotional load of stressful situations	1–5	3.35	1.06	-0.36	-0.56
24. I am able to sort out difficult problems when life presents those to me	1–5	3.41	0.94	-0.31	-0.31
11. I can tolerate frustrations of daily life	1–5	3.41	1.01	-0.35	-0.37
22. I can usually adapt myself to different contexts with no difficulties	1–5	3.39	0.99	-0.34	-0.39
26. When I feel an intense emotion, I can control it	1–5	3.24	1.09	-0.15	-0.75
**Factor 3: Relational attunement**					
28. I can easily attune to other people’s thinking	1–5	3.40	0.94	-0.34	-0.30
5. I can tune in other other people’s mental states	1–5	3.41	1.07	-0.39	-0.53
14. I’m able to empathize with others when they tell me something	1–5	3.90	0.98	-0.80	0.24
4. I’m able to get the deepest aspects of people around me	1–5	3.12	0.99	-0.12	-0.51
21. I am sensitive to what happens to others	1–5	3.48	1.04	-0.44	-0.37
**Factor 4: Relational discomfort**					
12. Others don’t understand me	1–5	2.94	1.19	0.11	-0.91
9. Relationships with other people prevent me from being myself	1–5	2.36	1.14	0.54	-0.61
27. People abandon me	1–5	2.38	1.26	0.55	-0.82
15. I am afraid to open up with other people	1–5	3.17	1.23	-0.09	-1.03
33. Some people are the cause of my problems	1–5	2.98	1.17	0.03	-0.86
**Factors 5: Distrust**					
13. It’s better to beware of others	1–5	3.43	1.07	-0.33	-0.58
29. It’s better to beware of strangers	1–5	3.64	1.04	-0.42	-0.53
20. I don’t trust others	1–5	2.93	1.16	0.11	-0.83
19. For me things are either white or black	1–5	2.32	1.22	0.67	-0.51
**Factors 6: Emotional dyscontrol**					
2. I am an impulsive person	1–5	2.93	1.16	0.11	-0.88
7. I sometimes feel like I am losing control of my emotions	1–5	2.64	1.27	0.30	-1.04
3. I sometimes experience mood swings I can’t control	1–5	2.90	1.30	0.05	-1.16
23. It happens to me to have conflicting emotions	1–5	3.14	1.06	-0.21	-0.60
**Other Psychological variables**					
FIMI score	1–4	3.02	0.61	− 0.37	− 0.11
Happiness-Depression score	1–4	2.23	0.74	0.27	− 0.75
Loneliness score	1–3	1.98	0.70	0.04	-1.30

SD = standard deviation

A general guideline for skewness is that if the number is greater than + 1 or lower than − 1, this is an indication of a substantially skewed distribution. For kurtosis, the general guideline is that if the number is greater than + 1, the distribution is too peaked

### Confirmatory Factorial Analysis (CFA) on the MMQ data

The assumptions of adequacy (KMO = 0.935) and sphericity (Bartlett’s test (ddl = 120) = 18607.60, *p* < 0.001) were met [39].

#### First CFA

The first CFA (model-1) tested the theoretical internal validity structure of the MMQ-English version.

The fit indices of model-1 were as follow: GFI = 0.89; NFI = 0.83; RFI = 0.82; IFI = 0.85; TLI = 0.84, CFI = 0.84; RMSEA = 0.059; SRMR = 0.072. Except for the RMSEA and the SRMR values, all these indices indicate that the model is not good. The standardized regression weights (factor loadings) were between 0.28 and 0.86, being statistically significant (*p* < 0.001). Particularly, as it can be seen in Table A (Appendix), five items had factor loading below the acceptable criteria (which stands at 0.50) (Hu & Bentler, 1999): Factor 1 - Reflexivity (Item1 = 0.45, Item8 = 0.49, Item31 = 0.48); Factor 5 - Distrust (Item19 = 0.28); Factor 6 - Emotional dyscontrol (Item2 = 0.40).

#### Second CFA

To improve the model, we deleted the five items with factor loadings below 0.50 and conducted a second CFA (model-2). The improved model (see path diagram in Fig. 1) metrics were as follow: GFI = 0.90; NFI = 0.86; RFI = 0.85; IFI = 0.88; TLI = 0.86; CFI = 0.88; RMSEA = 0.061; SRMR = 0.072. These indices indicate an improvement of the model-2 comparatively to the model-1. The GFI, RMSEA and the SRMR values suggest that the model-2 is not good but acceptable. The IFI and the CFI are very close to the acceptable threshold. In addition, the standardized regression weights (see Fig. 1) were between 0.50 and 0.87, being statistically significant (*p* < 0.001), which indicates high correlation between the items and their respective factors.

After examining the modification indices, we tried to improve the model-2 by establishing covariances between the standardized errors. This procedure was not successful: the model-2 did not significantly improve.

### Country (UK vs. USA) invariance tests

To find out whether the factor structure of the model-2 is invariant to country, a multi-group analysis was carried out from the model-2.

The configural invariance test showed an acceptable fit for the unconstrained model: GFI = 0.90; NFI = 0.87; RFI = 0.85; IFI = 0.89; TLI = 0.86; CFI = 0.90; RMSEA = 0.063; SRMR = 0.068. The metric invariance test indicated that the meaning of the six modeled constructs (factors) did not change across groups (X^2^ change = 14.23, *p* = 0.136).

### Internal reliability

Table 2 displays the main results of the internal reliability tests conducted for each subscale of the model-2 factorial structure.

**Table 2:** Reliability Statistics and Item-total Correlation: Model-2 (28 items*)

Factors / Items	Sub-scale Corrected Item-Total Correlation	Sub-scale Variance if Item Deleted	Sub-scale Cronbach’s Alpha if Item Deleted
**Factor 1: Reflexivity** **(Mean = 3.82 [SD = 0.59], Variance = 0.020, α = 0.80)**			
16. I ponder over what happens to me	0.49	13.117	0.79
18. I often think about why things happen	0.61	12.685	0.76
32. I’m keen on understanding why certain things happen to me	0.61	13.034	0.76
10. I’m interested in understanding my mental processes	0.61	12.539	0.76
17. I find beneficial to analyse my behaviour	0.59	12.594	0.76
6. Understanding what others feel is crucial in understanding their actions	0.41	14.339	0.80
**Factor 2: Ego-strength** **(Mean = 3.38 [SD = 0.76], Variance = 0.013, α = 0.85)**			
30. I am able to cope with difficult situations	0.72	14.81	0.81
25. I am able to bear the emotional load of stressful situations	0.72	14.23	0.81
24. I am able to sort out difficult problems when life presents those to me	0.65	15.51	0.82
11. I can tolerate frustrations of daily life	0.58	15.51	0.83
22. I can usually adapt myself to different contexts with no difficulties	0.51	16.18	0.85
26. When I feel an intense emotion, I can control it	0.60	14.95	0.83
**Factor 3: Relational attunement** **(Mean = 3.46 [SD = 0.74], Variance = 0.011, α = 0.79)**			
28. I can easily attune to other people’s thinking	0.60	9.37	0.74
5. I can tune in other other people’s mental states	0.64	8.54	0.72
14. I’m able to empathize with others when they tell me something	0.57	9.33	0.75
4. I’m able to get the deepest aspects of people around me	0.52	9.53	0.76
21. I am sensitive to what happens to others	0.49	9.46	0.77
**Factor 4: Relational discomfort** **(Mean = 2.76 [SD = 0.84], Variance = 0.014, α = 0.74)**			
12. Others don’t understand me	0.58	11.63	0.68
9. Relationships with other people prevent me from being myself	0.53	12.16	0.68
27. People abandon me	0.55	11.45	0.69
15. I am afraid to open up with other people	0.46	12.24	0.71
33. Some people are the cause of my problems	0.39	13.06	0.74
**Factors 5: Distrust** **(Mean = 3.34 [SD = 0.87], Variance = 0.13, α = 72)**			
13. It’s better to beware of others	0.55	3.51	0.60
29. It’s better to beware of strangers	0.56	3.57	0.59
20. I don’t trust others	0.49	3.42	0.68
**Factors 6: Emotional dyscontrol** **(Mean = 2.90 [SD = 1.02], Variance = 0.068, α = 0.76)**			
7. I sometimes feel like I am losing control of my emotions	0.70	4.10	0.62
3. I sometimes experience mood swings I can’t control	0.68	4.09	0.65
23. It happens to me to have conflicting emotions	0.51	5.66	0.82

SD = standard deviation; α = Cronbach’s alpha

Cronbach’s alpha: If the scale is an exploratory one, a good reliability is set at α > 0.7. If the scale is an established one, a good reliability is set at α > 0.80

Corrected Item-Total Correlation: A good corrected item-total correlation is set at *r*(correlation coefficient) > 0.30

Scale Variance if Item Deleted and Cronbach’s Alpha if Item Deleted corresponding values indicate the scale dimension (factor) variance and Cronbach’s alpha if the relevant item is deleted. These metrics suggest that no item should be deleted

*Comparatively to the original MMQ (see Table 1), items 1 (“I often try to explain what is happening to me”), item 8 (“I am able to reflect on my behaviours”), and item 31 (“I am a thoughtful person”) were removed from the Factor 1; the item 19 (“For me things are either white or black”) was removed from the Factor 2, and the item 2 (“I am an impulsive person”) was removed from the Factor 6

Indeed, in model-2, the Cronbach’s alpha coefficient shows that all MMQ subscales had acceptable internal reliability (≥ 70). The Corrected Item-total Correlation had *r* ≥ 0.30.

Regarding the model-1, see the internal reliability results in Appendix, Table B.

### Convergent validity

The correlation between the MMQ-English version total score and the FIMI total score (*r* = 21; *p* < 0.001; 95% confidence interval = 0.160 − 0.248; alpha level = 0.01) was significant, indicating good convergence of the two instruments.

### Correlations between the MMQ subscales

Table 3 displays the result of the bivariate correlations (Person) analysis between the MMQ subscales of the CFA model-2.

**Table 3:** Correlation matrix of the MMQ dimensions: Model-2 (28 items)

Factors	Reflexivity	Ego-strength	Relational attunement	Relational discomfort	Distrust	Emotional dyscontrol
Reflexivity	1	0.13^**^	0.50^**^	0.20^**^	0.21^**^	0.28^**^
Ego-strength	0.13^**^	1	0.30^**^	-0.32^**^	-0.12^**^	-0.40^**^
Relational attunement	0.50^**^	0.30^**^	1	-0.03	0.02	0.14^**^
Relational discomfort	0.20^**^	-0.32^**^	-0.03	1	0.52^**^	0.57^**^
Distrust	0.21^**^	-0.12^**^	0.02	0.52^**^	1	0.33^**^
Emotional dyscontrol	0.28^**^	-0.40^**^	0.14^**^	0.57^**^	0.33^**^	1

**Correlation is significant at the 0.01 level (2-tailed)

*Correlation is significant at the 0.05 level (2-tailed)

The relationships are statistically significant, except for the Distrust vs. Relation attunement and Relation discomfort vs. Relation attunement. All significant relationships are positive, except for Relational discomfort vs. Ego-strength, Distrust vs. Ego-strength, and Emotional dyscontrol vs. Ego-strength.

See same results regarding model-1 in Appendix, Table C.

### Correlation between MMQ factors (model-2) and the sociodemographic, psycho-cognitive, mental health, and socio-functional variables

Table 4 exhibits the correlations coefficients between the MMQ six factors (model-2) and the set of sociodemographic, psycho-cognitive, mental health, and socio-functioning variables.

**Table 4:** Correlations between the six MMQ factors (model-2, 28 items) and the sociodemographic, psycho-cognitive, and socio-functional variables

MMQ Factors / Demo and Psycho Measures	Sex	Age	RS	EL	SES	TM	HD	L
Reflexivity (MMQ F1)	0.14^**^	− 0.07^**^	− 0.04	0.08^**^	0.03	0.40^**^	0.02	0.13^**^
Ego-strength (MMQ F2)	− 0.11^**^	0.06^**^	0.04	0.04	0.13^**^	0.31^**^	− 0.48^**^	− 0.31^**^
Relational attunement (MMQ F3)	0.20^**^	− 0.01	0.00	0.05^*^	0.03	0.60^**^	− 0.12^**^	− 0.01
Relational discomfort (MMQ F4)	0.101^**^	− 0.07^**^	− 0.09^**^	− 0.06^**^	− 0.14^**^	− 0.21^**^	0.57^**^	0.60^**^
Distrust (MMQ F5)	0.03	0.00	0.02	− 0.10^**^	− 0.11^**^	− 0.19^**^	0.30^**^	0.29^**^
Emotional dyscontrol (MMQ F6)	0.09	0.91	0.24	0.00	0.00	0.00	0.00	0.00
Good MMQ (F1, F2, F3)	0.09^**^	− 0.01	0.00	0.08^**^	0.09^**^	0.58^**^	− 0.27^**^	− 0.11^**^
Poor MMQ (F4, F5, F)	− 0.16^**^	− 0.08^**^	− 0.03	− 0.08^**^	− 0.14^**^	− 0.21^**^	0.54^**^	0.54^**^

MMQ = Multidimensional Mentalizing Questionnaire; Demo = demographic; Psycho = psychological; F = factor; Sex (1 = male; 2 = female); Age (in terms of years); RS = relationship status (1 = not in relationship; 2 = in relationship); EL = education level (years of schooling); SES = socio-economic status (1 = low; 2 = intermediate; 3 = high); TM = theory of mind score; HD = happiness-depression score; L = loneliness score

**Correlation is significant at the 0.01 level (2-tailed)

*Correlation is significant at the 0.05 level (2-tailed)

Reflexivity (F1[Factor 1]) was significantly and positively correlated with *sex*, with *education level*, with *SES*, and with *loneliness*; it was negatively correlated with *age*. Ego-strength (F2) was significantly and positively associated with *age*, with *SES*, and with the *theory of mind* score; it was negatively associated with *sex*, with *happiness-depression*, and with *loneliness*. Relational attunement (F3) was significantly and positively correlated with *sex*, with *education level*, and with SES; it was negatively correlated with *happiness-depression* score. Relational discomfort (F4) was significantly and positively associated with *sex*, with *happiness-depression* score, and with *loneliness*; it was negatively associated with *age*, with *relationship status*, with *educational level*, with *SES*, and with the *theory of mind* score. Distrust (F5) was significantly and positively correlated with *happiness-depression* scores and with *loneliness*; it was negatively correlated with *education level*, with *SES*, and with the *theory of mind* score. Emotional dyscontrol (F6) was significantly correlated with none of the demographic and psychological variables. Finally, good mentalizing (calculated by averaging F1, F2, and F3) was significantly and positively associated with *sex*, with *education level*, with *SES*, and with the *theory of mind* score; it was negatively associated with the *happiness-depression* score, and with loneliness. Poor mentalizing (calculated by averaging F4, F5, and F6) was significantly and positively correlated with *sex*, with the *happiness-depression* score, and with *loneliness*; it was negatively associated with *age*, education level, with *SES*, and with the *theory of mind* score.

## Discussion

The present study aimed at (a) testing the validity of the MMQ-English version factor structure and its psychometric properties, and (b) to explore the relationships between the six dimensions of the MMQ and a set of sociodemographic, psycho-cognitive, mental health, and socio-functional variables.

### Factor structure and psychometric properties

The MMQ-English version six factorial structure was validated with acceptable/satisfactory goodness of fit, after applying improvement techniques on the initial CFA model. These improvement techniques required the elimination of three, one, and one items respectively from the Factor (F)1 (reflexivity), F5 (distrust), and F6 (emotional dyscontrol) of the initial factor structure. Thus, the validated English version has a total of 28 items, compared to 33 items in the original MMQ [22, 23] factor structure, validated in the Italian sample. Indeed, comparatively to the original MMQ (see Table 1), the items 1 (“I often try to explain what is happening to me”), the item 8 (“I am able to reflect on my behaviors”), and item 31 (“I am a thoughtful person”) were removed from Factor 1; the item 19 (“For me things are either white or black”) was removed from Factor 2, and the item 2 (“I am an impulsive person”) was removed from Factor 6. Regarding the psychometric properties, the current study findings indicated that: (a) all six factors of the MMQ-English version have acceptable internal reliability (Cronbach alpha [α] ≥ 70; corrected item-total correlation [r] ≥ 0.30); (b) the correlations between all items and their respective dimensions are significant (*p* < 0.001), and the standardized regression weights between the items and their respective dimensions are above the threshold (≥ 0.50); (c) the correlation between the MMQ-English version total score and the FIMI total score was significant, indicating good convergence between the two instruments. The difference between the MMQ-English version and the original MMQ-Italian version may stem from the differences on the sample used. As mentioned in the introduction, the original MMQ version was validated among a relatively homogenous Italian sample, the large majority of whom were female. Comparatively, the validity of the MMQ-English version was tested on a larger sample (1823 participants), diverse in terms of sex, education level, and country of origin. The sample discrepancies might also explain why the original version [22, 23] showed a relatively better goodness of fit (RMSEA = 0.053; SRMR = 0.067; TLI = 0.90; CFI = 0.90) compared with the indices of the present version (RMSEA = 0.061; SRMR = 0.072; TLI = 0.86; CFI = 0.90). While the structure was invariant across two countries (UK vs. USA) representing 95% of the sample, overall, these findings, when compared to the finding of Gori et al. [22, 23], suggest that MMQ is susceptible to socio-cultural and/or cross-cultural differences and that the factor structure need to be improved in future studies, which must have cross-cultural design.

### Good vs. bad mentalizing

As stressed in the introduction section, the integrated and multilevel model of mentalizing proposed in the MMQ articulates the idea of good and bad mentalizing throughout four different axes, corresponding to four polarities: explicit-implicit, outside-inside, self-other, and cognitive-affective [22]. These four axes can be assimilated to the four dimensions of mentalizing in the Fonagy’s model [40], also conceived in terms of duel and opposite aspects: automatic-controlled, internal-external, self-other, and cognitive-affective. The main idea of this model is that good mentalizing requires the ability of using these four polarities dynamically, with flexibility, balancing them appropriately as function of the needs of the circumstances, the situation, and the social environment. This perspective might give insight into the findings of the present study, regarding the correlations among the six MMQ factors, and the correlations between the six factors and the sociodemographic, psycho-cognitive, mental health, and socio-functional variables.

### Correlations among the six MMQ mentalizing factors

In this study, in general, the three dimensions associated with good mentalizing (reflexibility, ego-strength, relational attunement) are significantly and negatively associated with the three dimensions associated to bad mentalizing (relational discomfort, distrust emotional dyscontrol). This finding further confirms and validated the model of mentalizing proposed in the MMQ. However, there is some exceptions, among them, relational attunement vs. relational discomfort was not significantly correlated; relational attunement vs. distrust was not significantly correlated neither. Results from the original MMQ version validation [22] showed non-significant correlations between reflexibility vs. emotional dyscontrol and between relational attunement vs. emotional dyscontrol. Considering, for instance, that in the MMQ model of bad and good mentalizing the relational attunement and the relational discomfort are opposite poles, it seems anti-hypothetic (counter-intuitive?) that, in the present study and in the study by Gori et al. [22], they are not significantly and negatively correlated. Nevertheless, the overall differences between the two studies suggest that the referred non-significance is probably a result of the variance within and between the samples, rather than indication of a “defect” concerning the conceptual model itself. In any case, this is an issue warranting further investigation in future studies.

### Associations with sociodemographic and psychological variables

#### Significant associations

As for the correlational relationship between the six dimensions of the validated MMQ-English version (model-2) and the set of sociodemographic and psychological variables, generally, the results of the present study are in line with the previous studies.

For instance, the overall findings of the present study suggest that female were significantly more likely to have good mentalizing than their male counterparts. According to the integrative and multilevel model of mentalizing [22, 23] (see Fig. 1) in which the MMQ is based, good mentalizing is achieved when an individual has high scores on the reflexivity, ego-strength, and relational attunement dimensions, which leads to greater internal-external openness abilities and, eventually, to a better cognitive-effective integration. In line with this particular result, findings from magnetic resonance imaging (MRI)-based study [41] suggested that women may have better neuro-cognitive capabilities for mentalizing than men. Furthermore, some research works [42, 43] have indicated that female have better mentalizing performances than their male counterparts and that this difference can be explained as follows: (a) women are generally more motivated to engage in deeper social relationships and tend to socialize more, better, and in much more diverse contexts than men; (b) women, compared to men, are less limited by language abilities in their efforts to mentalize. Thus, it seems that distinct neuro-psychological attributes/functioning and social skills, resulting from specific patterns of socialization, lead to significant sex differences regarding mentalizing performances.

While some studies [42] have shown that, from childhood to adulthood (7–18 years-old), mentalizing gets better as the age advances, in the current study, the overall findings do not indicate that from 19 to 76 years-old, the age has significant effect on participants’ mentalizing performances. There is indication, however, that the reflexivity dimension decline as age advances. This result can be linked with studies [44] indicating that as people aged, they tend to lose cognitive flexibility, because they tend to be more affected by *bias through feedback* than young individuals, i.e., as their ability to acquire and updating information decline, older people tend to over-rely on their past experiences, instead of giving more weight to the situational indices.

More educated people are more likely to have good mentalizing than less educated people. Individuals with greater SES are more likely to have good mentalizing than those with lower SES. These findings can be explained by the fact that education improves both general and specific cognitive abilities, and greater SES comes with the opportunity to experience diverse social environments and interpersonal/intergroup relationships as well as to have better, diversified, and longer formal and informal education [45].

People higher on theory of mind abilities tend to be also higher on good mentalizing compared to those with lower theory of mind capacities. This result confirms several previous works demonstrating the significant positive relationship between the scores on mentalizing and theory of mind [3]. In fact, as emphasized in the introduction section, these two concepts are highly related which underlines the fair convergent validity of the MMQ in the current study.

Depressed individuals are more likely to have poor mentalizing as opposed to happier individuals. According to the integrative and multilevel model of mentalizing [22, 23] in which the MMQ is based, poor mentalizing is achieved when an individual has high scores on the relational discomfort, distrust, and emotional dyscontrol dimensions, which leads to greater internal-external closure and, eventually, to cognitive-effective split. This particular result is backed by previous studies [46], which elaborated that depressed mood leads to increases in arousal and stress levels, resulting in impairments and distortions in mentalizing, which in turn may contribute to the development or persistence of depressive symptoms.

Individuals experiencing higher scores on loneliness were more likely to have poor mentalizing compared to people feeling satisfied with their social relationships. Previous studies have found negative correlation between loneliness and mentalizing only in patients suffering from schizophrenia [47]. The present study, conducted on non-clinical population, suggests that this relationship exists also among individuals from the general population. Lonely persons are significantly more affected by anxiety, depression, and impairments in cognition and social functioning [48], which may explain why they have higher probability of having poor mentalizing.

Overall, the findings discussed above highlight the importance of education and mentalizing training to improve mental health and wellbeing among the general population.

#### Non-significant associations

In the present study, the relationship between reflexivity (MMQ F1) and happiness/depression was no significant. Reflexivity can be defined as “the fact of someone being able to examine their own feelings, reactions, and motives (reasons for acting) and how these influence what they do or think in a situation” [49]. The concept of reflexivity, as define above, is related to the psychological concept of mindfulness, define as “awareness of one’s internal states and surroundings” which “can help people avoid destructive or automatic habits and responses by learning to observe their thoughts, emotions, and other present-moment experiences without judging or reacting to them” [50]. Several studies have shown that mindfulness is connected to positive outcomes related to mental health and well-being and that this association is mediated by the individual purpose in life and behavioral activation [51]. Thus, it would be interesting, in future studies, to further investigate the relationship between reflexivity and happiness/depression by including possible mediators such as the purpose in life or the behavioral activation.

The association between relational attunement (MMQ F3) and loneliness was also no significant. Relational attunement is “a kinesthetic and emotional sensing of others, knowing their rhythm, affect and experience by metaphorically being in their skin” [52]. As the ability to feel what the other person is feeling by entering their inner world, cognitive and emotional attunement is critical in all relationships while being an essential component of friendships and romantic partnerships [52]. Accordingly, previous studies have shown a significant and negative relationship between relational attunement and loneliness [53]. In the current study, it is possible that some unknown factors moderate/mediate this association. Thus, it is an issue that must be investigated thoroughly in future studies.

Finally, based in previous studies [52, 53], it could have been hypothesize an statistical significant association between emotional dyscontrol (MMQ F6), happiness/depression, and loneliness. The finding of the present study indicated, however, that it was not the case; the relationship was not significant. As mention above, one of the best ways to address this “contradiction” in future studies is to design a methodology that includes possible moderators/mediators and/or conduct cluster analyses to group participants into different categories as function of given variables (sociodemographic characteristics, personality traits, social experiences, etc.) and run association analyses between the chosen variables (e.g., emotional dyscontrol and happiness) within each cluster to see what can come out.

### Limitations

The present study involves a cross sectional assessment. Overall, 95% of the participants come from just two countries, the UK and the USA. Furthermore, the sample included is not a representative sample of the population. Further studies may assess mentalizing across different clinical and representative samples within longitudinal design. The study however included a sex-balanced large sample from different countries. Finally, this study does not give a response to the question discussed in the introduction, which can be reformulated here as follow: to what extent MMQ assesses people’s mentalizing abilities (and not only their beliefs about their mentalizing abilities)? As discussed recently by Wendt et al. [25, p. 9–10], to validated a “mindreading” instrument in terms of assessing “abilities” and not just “beliefs about one’s own abilities”, future studies must go behind the classic tests of internal consistency, structural validity, or convergent validity (with another self-reported measurement instrument), and create task-based assessment tool to be used as “gold standard” to validate a given self-report instrument capacity to assess a set of “real” abilities. In this case, a “gold standard” to validated the MMQ capacity to assess “real” mentalizing abilities must contain tasks in line with the functionalist reconceptualization of mentalizing abilities, that is, mentalizing abilities viewed “in terms of its ecological utility in everyday life, such as the ability to achieve favorable outcomes in interpersonal situations” in which mentalizing is required; appropriated tasks must be designed specifically to go behind inference “of mental states from static information to a broader perspective encompassing interpersonal communication” [25, p. 11–12].

## Conclusion

The present study allowed the validation of the 28-items MMQ-English version. The current study results indicated that male, less educated, lower SES, depressed, and lonely individuals are significantly more likely to have poor mentalizing compared with female, highly educated, greater SES, happier, non-lonely individuals. Finally, these findings emphasize the need to consider mentalizing as a multifaceted and multilevel psychological construct centrally involved in a variety of human psycho-cognitive expressions, behavior, and mental health conditions.

### Electronic supplementary material

Below is the link to the electronic supplementary material.

Supplementary Material 1

## Data Availability

The data used in this study are available at: https://gitlab.huma-num.fr/gveracruz/social-cognition/-/blob/main/Data/data_study_MMQ.xlsx.

## Abbreviations

BPD: Borderline personality disorder
MBT: Mentalization-based therapy
AT: Affect Task
MSA: Mentalizing Stories for Adolescents
RFS: Reflective Functioning Scale
MAS: Metacognition Assessment Scale
AAI: Adult Attachment Interview
RFQ: Reflective Functioning Questionnaire
MZQ: Mentalization Questionnaire
MIS: Mentalization Imbalances Scale
MentS: Mentalization Scale
IMQ: Interactive Mentalizing Questionnaire
MMQ: Multidimensional Mentalizing Questionnaire
FIMI: Four-Items Mentalisation Index
SDHS: Short Depression-Happiness Scale
UCLA 3-Item: University of California Los Angeles Short Form Loneliness Scale
CFA: Confirmatory Factorial Analysis
KMO: Kaiser–Meyer–Olkin test
CFI: Comparative Fit Index
TLI: Tucker Lewis Fit Index
SRMR: Standardized Root Mean Square Residual
RMSEA: Root Mean Square Error of Approximation
F: Factor
RS: Relationship status
EL: Education level
SES: Socio-economic status
TM: Theory of mind score
HD: Happiness-depression score
L: Loneliness score
