# Supplementary material for: Validation of the english version of the Multidimensional Mentalizing Questionnaire (MMQ)
Source: BMC Psychol. 2024 Jun 12;12:344. doi: 10.1186/s40359-024-01837-z (PMC11167805; doi:10.1186/s40359-024-01837-z)
Supplement: Supplementary file 1 — Supplementary Material 1 [file 40359_2024_1837_MOESM1_ESM.docx]

**APPENDIX**

**Table A.**

The standardized regression weights (factor loadings): Model-1

| Factors / Items | Factor loadings |
| --- | --- |
|  |  |
| **Factor 1: Reflexivity** |  |
| 1. I often try to explain what is happening to me* | 0.45 |
| 16. I ponder over what happens to me | 0.54 |
| 18. I often think about why things happen | 0.67 |
| 32. I’m keen on understanding why certain things happen to me | 0.69 |
| 10. I’m interested in understanding my mental processes | 0.68 |
| 17. I find beneficial to analyse my behaviour | 0.65 |
| 31. I am a thoughtful person * | 0.48 |
| 8. I am able to reflect on my behaviours* | 0.49 |
| 6. Understanding what others feel is crucial in understanding their actions | 0.54 |
|  |  |
| **Factor 2: Ego-strength** |  |
| 30. I am able to cope with difficult situations | 0.79 |
| 25. I am able to bear the emotional load of stressful situations | 0.79 |
| 24. I am able to sort out difficult problems when life presents those to me | 0.70 |
| 11. I can tolerate frustrations of daily life | 0.66 |
| 22. I can usually adapt myself to different contexts with no difficulties | 0.56 |
| 26. When I feel an intense emotion, I can control it | 0.67 |
|  |  |
| **Factor 3: Relational attunement** |  |
| 28. I can easily attune to other people’s thinking | 0.71 |
| 5. I can tune in other people’s mental states | 0.74 |
| 14. I’m able to empathize with others when they tell me something | 0.64 |
| 4. I’m able to get the deepest aspects of people around me | 0.63 |
| 21. I am sensitive to what happens to others | 0.55 |
|  |  |
| **Factor 4: Relational discomfort** |  |
| 12. Others don’t understand me | 0.69 |
| 9. Relationships with other people prevent me from being myself | 0.60 |
| 27. People abandon me | 0.66 |
| 15. I am afraid to open up with other people | 0.57 |
| 33. Some people are the cause of my problems | 0.50 |
|  |  |
| **Factors 5: Distrust** |  |
| 13. It’s better to beware of others | 0.64 |
| 29. It’s better to beware of strangers | 0.62 |
| 20. I don’t trust others | 0.73 |
| 19. For me things are either white or black* | 0.28 |
|  |  |
| **Factors 6: Emotional dyscontrol** |  |
| 2. I am an impulsive person* | 0.40 |
| 7. I sometimes feel like I am losing control of my emotions | 0.86 |
| 3. I sometimes experience mood swings I can’t control | 0.80 |
| 23. It happens to me to have conflicting emotions | 0.59 |

*Items with factor loading <.50. These items were dropped prior to the second Confirmatory Factorial Analysis (CFA).

**Table B.**

Reliability Statistics and Item-total Correlation: Model-1

| Factors / Items | Sub-scale Corrected Item-Total Correlation | Sub-scale Variance if Item Deleted | Sub-scale Cronbach's Alpha if Item Deleted |
| --- | --- | --- | --- |
|  |  |  |  |
| **Factor 1: Reflexivity**  **(Mean=3.78 [SD=0.63], Variance = 0.028, α = 0.81)** |  |  |  |
| 1. I often try to explain what is happening to me * | 0.39 | 26.69 | 0.81 |
| 16. I ponder over what happens to me | 0.48 | 26.31 | 0.80 |
| 18. I often think about why things happen | 0.60 | 25.61 | 0.78 |
| 32. I’m keen on understanding why certain things happen to me | 0.62 | 25.93 | 0.78 |
| 10. I’m interested in understanding my mental processes | 0.61 | 25.28 | 0.78 |
| 17. I find beneficial to analyse my behaviour | 0.60 | 25.35 | 0.78 |
| 31. I am a thoughtful person* | 0.41 | 28.05 | 0.81 |
| 8. I am able to reflect on my behaviours* | 0.44 | 27.45 | 0.81 |
| 6. Understanding what others feel is crucial in understanding their actions | 0.45 | 27.44 | 0.80 |
|  |  |  |  |
| **Factor 2: Ego-strength**  **(Mean=3.38 [SD=0.76], Variance = 0.013, α = 0.85)** |  |  |  |
| 30. I am able to cope with difficult situations | 0.72 | 14.81 | 0.81 |
| 25. I am able to bear the emotional load of stressful situations | 0.72 | 14.23 | 0.81 |
| 24. I am able to sort out difficult problems when life presents those to me | 0.65 | 15.51 | 0.82 |
| 11. I can tolerate frustrations of daily life | 0.58 | 15.51 | 0.83 |
| 22. I can usually adapt myself to different contexts with no difficulties | 0.51 | 16.18 | 0.85 |
| 26. When I feel an intense emotion, I can control it | 0.60 | 14.95 | 0.83 |
|  |  |  |  |
| **Factor 3: Relational attunement**  **(Mean=3.46 [SD=0.74], Variance = 0.011, α = 0.79)** |  |  |  |
| 28. I can easily attune to other people’s thinking | 0.60 | 9.37 | 0.74 |
| 5. I can tune in other people’s mental states | 0.64 | 8.54 | 0.72 |
| 14. I’m able to empathize with others when they tell me something | 0.57 | 9.33 | 0.75 |
| 4. I’m able to get the deepest aspects of people around me | 0.52 | 9.53 | 0.76 |
| 21. I am sensitive to what happens to others | 0.49 | 9.46 | 0.77 |
|  |  |  |  |
| **Factor 4: Relational discomfort**  **(Mean=2.76 [SD=0.84], Variance = 0.014, α = 0.74)** |  |  |  |
| 12. Others don’t understand me | 0.58 | 11.63 | 0.68 |
| 9. Relationships with other people prevent me from being myself | 0.53 | 12.16 | 0.68 |
| 27. People abandon me | 0.55 | 11.45 | 0.69 |
| 15. I am afraid to open up with other people | 0.46 | 12.24 | 0.71 |
| 33. Some people are the cause of my problems | 0.39 | 13.06 | 0.74 |
|  |  |  |  |
| **Factors 5:** **Distrust**  **(Mean=3.08 [SD=0.78], Variance = 0.032,** **α = 65)** |  |  |  |
| 13. It’s better to beware of others | 0.49 | 5.99 | 0.52 |
| 29. It’s better to beware of strangers | 0.50 | 6.07 | 0.51 |
| 20. I don’t trust others | 0.49 | 5.67 | 0.51 |
| 19. For me things are either white or black* | 0.21 | 6.91 | 0.72 |
|  |  |  |  |
| **Factors 6:** **Emotional dyscontrol**  **(Mean=2.90 [SD=0.92], Variance = 0.068, α = 0.76)** |  |  |  |
| 2. I am an impulsive person* | 0.39 | 9.40 | 0.79 |
| 7. I sometimes feel like I am losing control of my emotions | 0.67 | 7.13 | 0.63 |
| 3. I sometimes experience mood swings I can’t control | 0.67 | 7.27 | 0.63 |
| 23. It happens to me to have conflicting emotions | 0.50 | 9.15 | 0.73 |

*Items with factor loading ≤.50. These items were dropped prior to the second Confirmatory Factorial Analysis (CFA).

SD = standard deviation; α **=** Cronbach’s alpha.

Cronbach’s alpha: If the scale is an exploratory one, a good reliability is set at **α** ≥ 0.7. If the scale is an established one, a good reliability is set at α ≥ 0.80.

Corrected Item-Total Correlation: A good corrected item-total correlation is set at *r*(correlation coefficient) ≥ 0.30.

Scale Variance if Item Deleted and Cronbach's Alpha if Item Deleted corresponding values indicate the scale dimension (factor) variance and Cronbach’s alpha if the relevant item is deleted. These metrics suggest that no item should be deleted.

**Table C.**

**C**orrelation matrix of the MMQ dimensions: Model-1

| **Factors** | Reflexivity | Ego-strength | Relational attunement | Relational discomfort | Distrust | Emotional dyscontrol |
| --- | --- | --- | --- | --- | --- | --- |
| Reflexivity | 1 | 0.19^**^ | 0.56^**^ | 0.14^**^ | 0.11^**^ | 0.25^**^ |
| Ego-strength | 0.19^**^ | 1 | 0.30^**^ | -0.32^**^ | -0.11^**^ | -0.35^**^ |
| Relational attunement | 0.56^**^ | 0.30^**^ | 1 | -0.03 | -0.01 | 0.14^**^ |
| Relational discomfort | 0.14^**^ | -0.32^**^ | -0.03 | 1 | 0.53^**^ | 0.55^**^ |
| Distrust | 0.11^**^ | -0.11^**^ | -0.01 | 0.53^**^ | 1 | 0.32^**^ |
| Emotional dyscontrol | 0.25^**^ | -0.35^**^ | 0.14^**^ | 0.55^**^ | 0.32^**^ | 1 |

**Correlation is significant at the 0.01 level (2-tailed).

*Correlation is significant at the 0.05 level (2-tailed).
